# Influence of Heavy Metal Stress on Antioxidant Status and DNA Damage in *Urtica dioica*

**DOI:** 10.1155/2013/276417

**Published:** 2013-06-04

**Authors:** Darinka Gjorgieva, Tatjana Kadifkova Panovska, Tatjana Ruskovska, Katerina Bačeva, Trajče Stafilov

**Affiliations:** ^1^Faculty of Medical Sciences, Goce Delčev University, Krste Misirkov Street bb, P.O. Box 201, 2000 Štip, Macedonia; ^2^Faculty of Pharmacy, Ss. Cyril and Methodius University, 1000 Skopje, Macedonia; ^3^Institute of Chemistry, Faculty of Natural Sciences and Mathematics, Ss. Cyril and Methodius University, 1000 Skopje, Macedonia

## Abstract

Heavy metals have the potential to interact and induce several stress responses in the plants; thus, effects of heavy metal stress on DNA damages and total antioxidants level in *Urtica dioica* leaves and stems were investigated. The samples are sampled from areas with different metal exposition. Metal content was analyzed by Inductively Coupled Plasma-Atomic Emission Spectrometer (ICP-AES), for total antioxidants level assessment the Ferric-Reducing Antioxidant Power (FRAP) assay was used, and genomic DNA isolation from frozen plant samples was performed to obtain DNA fingerprints of investigated plant. It was found that heavy metal contents in stems generally changed synchronously with those in leaves of the plant, and extraneous metals led to imbalance of mineral nutrient elements. DNA damages were investigated by Random Amplified Polymorphic DNA (RAPD) technique, and the results demonstrated that the samples exposed to metals yielded a large number of new fragments (total 12) in comparison with the control sample. This study showed that DNA stability is highly affected by metal pollution which was identified by RAPD markers. Results suggested that heavy metal stress influences antioxidant status and also induces DNA damages in *U. dioica* which may help to understand the mechanisms of metals genotoxicity.

## 1. Introduction

Metals constitute one of the major groups of genotoxic environmental pollutants possessing serious threat to human as well as environmental well-being. Heavy metal stress in all living organisms often results in the production of reactive oxygen species (ROS), which are relatively reactive compared to molecular oxygen and thus potentially toxic [1, 2]. 

Tolerance to heavy metal stress has been correlated with efficient antioxidative defense system, as shown by many authors [2–4]. Among different present methods used to assess the total antioxidant capacity of plants, one of them is the Ferric-Reducing Antioxidant Power (FRAP) assay of Benzie and Strain [5]. 

Heavy metals also induce several cellular stress responses and damage to different cellular components such as membranes, proteins, and DNA. Recently, advances in molecular biology have led to the development of a number of selective and sensitive assays for DNA analysis in ecogenotoxicology. DNA-based techniques, like Random Amplified Polymorphic DNA (RAPD), is used to evaluate the variation at the DNA level, and differences can clearly be shown when comparing DNA fingerprints from individuals exposed and/or nonexposed to genotoxic agents [6–10].

Monitoring the pollution status of the environment using plants is one of the main topics of environmental biogeochemistry [11]. Although heavy metals are naturally present in soils, contamination comes from different sources, mostly industry (mainly nonferrous, iron and steel, and chemical industries), waste incineration, agriculture (use of polluted waters for irrigation, fertilizers, and phosphates, especially, pesticides containing heavy metals), combustion of fossil fuels, and traffic [12].

Nettle, (*Urtica dioica*, Urticaceae) was chosen as the object of this study because it is a widespread plant in R. Macedonia, edible, used in medicinal purposes, and also frequently used as a model plant in different studies [13–15]. The objective of the present study was to investigate; thus, exposure to different metals can induce direct DNA damage and significant changes in metal content in the plant and also endogenous total antioxidants level.

## 2. Materials and Methods

### 2.1. Sampling Area

**Figure 1:**
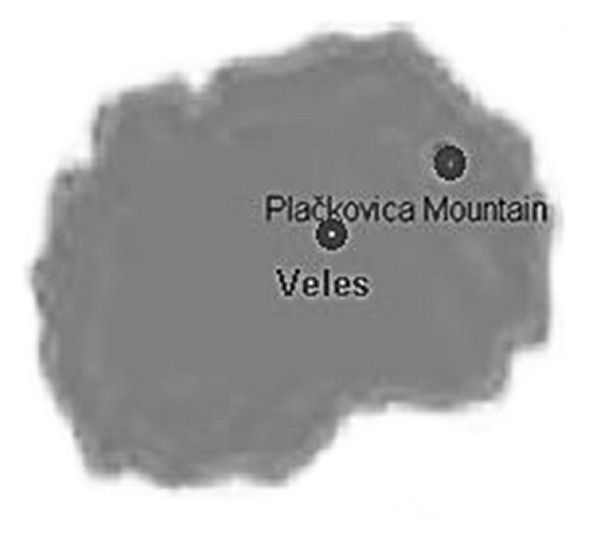
City of Veles and Plačkovica Mountain as sampling areas.

Plant samples from the industrialized area were taken from 10–100 m around the lead and zinc smelting plant “MHK Zletovo” in Veles area, while for uncontaminated controls, samples were taken from Plačkovica Mountain, about 60 km from the city of Veles (Figure 1). Leaves and stems from plants were analyzed. The plants were identified and specimens are deposited at the Department of Pharmacognosy, Faculty of Pharmacy, Skopje, Republic of Macedonia. Element analysis, FRAP analysis, and DNA extraction were performed.

### 2.2. Sample Preparation for Element Analysis

All plant samples, not rinsed, were air dried, milled in a nonmetal micro-hammer, and stored in clean paper bags. 0.5 g was weighed and placed into PTFE vessels with 5 mL HNO_3_ (69% Merck, Tracepur) and 2 mL H_2_O_2_ (30%, m/V; Merck); mixture was digested by microwave (MARS CEM XP 1500) with two steps procedure at 180°C. Digests were filtered on filter paper (Munktell), quantitatively transferred in 25 mL calibrated flasks, diluted with demineralized water, and analyzed by inductively coupled plasma-atomic emission spectrometer (ICP-AES), Varian 715-ES, for selected metals. Standards of selected metals were set by dilution of stock standards which were prepared using analytical grade salts of metals (Merck Multielement standard 1000 mg/L). Samples were analyzed in triplicate. All results were calculated on a dry weight basis (mg kg^−1^ dw).

### 2.3. FRAP Assay

The total antioxidant power of a freshly prepared, cooled, and filtered infusion (5 g of dry leaves or stems/100 mL of boiling, distilled water) of each sample was measured using the FRAP assay. In the FRAP assay, reductants (antioxidants) in the sample reduce Fe^3+^/tripyridyltriazine complex, present in stoichiometric excess, to the blue colored ferrous form, with an increase in absorbance at 595 nm. Samples were analyzed using microplate reader (ChemWell) at 600 nm. The antioxidant status is expressed as *μ*mol FeSO_4_ L^−1^. All values are means of triplicate analyses ± SD.

### 2.4. Genomic DNA Isolation

Frozen plant samples were used for DNA isolation. 0.5 to 0.7 cm disks of leaf tissue were catted with standard one-hole paper punch. Samples were kept on ice, while the procedure was done. DNA extractions were performed using REDExtract-N-Amp Plant PCR Kit (Sigma-Aldrich) following the instructions of the manufacturer. Plant disk was placed into a 1.5 mL microcentrifuge tube with 100 *μ*L extraction solution and incubated for 10 minutes at 95°C. 100 *μ*L of dilution solution is added and vortexes. Extract is stored at 2–8°C until use.

### 2.5. RAPD Amplification Methods

PCR reactions were performed using REDExtract-N-Amp Plant PCR Kit (Sigma-Aldrich). PCR reactions were performed in reaction mixtures of 20 *μ*L containing 10 ng of genomic DNA, 0.4 *μ*M primer (Sigma-Aldrich), and 10 *μ*L REDExtract-N-Amp PCR reaction mix. The REDExtract-N-Amp PCR reaction mix is a ready mix containing buffer, salts, dNTPs, and REDTaq DNA polymerase. Sequences (5′ → 3′) from primer 1 to 7 (with 60–70% GC content) used are GGTGCGGGAA (P1); GTTTCGCTCC (P2); GTAGACCCGT (P3); AAGAGCCCGT (P4); AACGCGCAAC (P5); CCCGTCAGCA (P6); GGCACTGAGG (P7), respectively. Amplifications were performed in a DNA thermocycler (Mastercycler personal, Eppendorf) programmed for 5 min at 95°C (initial denaturing step), 45 consecutive cycles each consisting of 1 min at 95°C (denaturing), 1 min at 36°C (annealing), 2 min at 72°C (extension), and followed by the last cycle for 5 min at 72°C (final extension step). Negative controls with water, without any template DNA, were always included to monitor for contamination. After amplification, electrophoresis of RAPD reaction products was performed in 2% (w/v) agarose (Agarose 1000; Invitrogen) using a TBE (Tris/borate/EDTA) buffer system (1 × TBE = 90 mM tris base, 90 mM boric acid, and 2 mM EDTA). DNA bands were stained with ethidium bromide for 10 minutes, visualized, and photographed under UV light (Biometra). All amplifications were repeated twice in order to confirm the reproducible amplification of scored fragments. Only reproducible and clear amplification bands were scored for the construction of the data matrix.

## 3. Results and Discussion

Veles area (around lead and smelting plant) was chosen as an investigated area because it is an important source of lead and zinc pollution in R. Macedonia, with estimated lead emission of 83 tons per year according to the National Environmental Action Plan (NEAP) [16], and there were several investigations in the region of Veles for heavy metals contents [17–20]. As shown in Table 1, varying amounts of metal contents were noted not only for the heavy metals, but also for essential metals. Levels of metals uptake and accumulation by plants increased with increasing metal concentration in environment.

Results for total antioxidants level in samples sampled from two different areas obtain with FRAP assay are presented in Table 2. 

**Figure 2:**
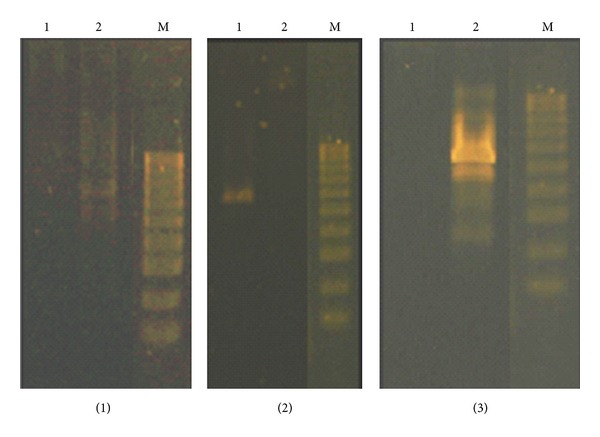
RAPD profiles of *U. dioica* sampled from two different areas obtained with different primers ((1) primer 1, (2) primer 2, and (3) primer 3): (1) *U. dioica* Plačkovica and (2) *U. dioica* Veles; M is DNA marker.

**Figure 3:**
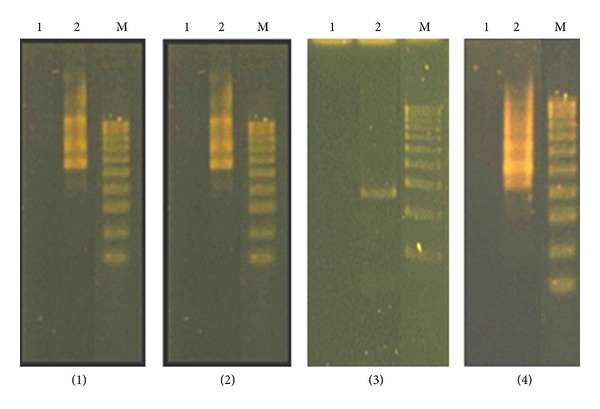
RAPD profiles of *U. dioica* sampled from two different areas obtained with different primers ((1) primer 4, (2) primer 5, (3) primer 6, and (4) primer 7): (1) *U. dioica* Plačkovica and (2) *U. dioica* Veles; M is DNA marker.

RAPD profile generated by samples exposed to heavy metals was different from those obtained using control DNA. The RAPD profiles are presented in Figures 2 and 3. A summary of results obtained with the primer set used with control and toxic metals exposed DNA samples is shown in Table 3. Polymorphism (*P*, in %) was calculated as the following:

(1) $$\begin{array}{c} P\;=\;\left[\frac{a+b}{c}\right]\cdot 100, \end{array}$$

where *a* is the number of new bands, detected in samples (different from the control), *b* is the number of disappeared bands and *c* is the total number of scored bands. Polymorphism in RAPD profiles included disappearance of a normal band and appearance of a new band in comparison to the control.

Content of 102.2 mg kg^−1^ of Pb was observed in *U. dioica* leaves in samples taken from the area around the lead and zinc smelting plant. These results are corresponding to the fact that most uptake of Pb has been demonstrated to be through the leaves and fact that various authors [21, 22] refer to *U. dioica* as a plant possess a high natural potential for hyperaccumulation and hypertolerance of lead. *U. dioica* stems from the same location showed also high Pb content (24.79 mg kg^−1^). In contrast, in control plants sampled from Plačkovica Mountain, a content of 3.73 mg kg^−1^ was measured in plant leaves, which is in the normal range for Pb in plants, 0.1–10 mg kg^−1^ dw, according to Kabata-Pendias and Pendias [23], while generally, toxic concentrations of Pb are defined as 30–300 mg kg^−1^ [24].

Also extremely high values are determined for Zn content in *U. dioica* leaves from the Veles region. Zinc is an essential element in all organisms and is not considered to be highly phytotoxic, where toxicity limit for Zn (300–400 mg kg^−1^) depends on the plant species as well as on the growth stage [23]. According to the above mentioned criteria, investigated plants in this area are exposed to highly phytotoxic doses of Pb and Zn. 

This is valid also for Cd, which is very toxic metal and as far as is known, Cd is not a constituent of any metabolically important compound. Obtained values for Cd content (7.34 and 6.67 mg kg^−1^) in *U. dioica* leaves and stems, respectively, are also in the phytotoxic range. The normal limits of Cd content in plants are between 0.2–0.8 mg kg^−1^, and toxic concentrations of Cd are in the range of  5–30 mg kg^−1^ [23, 24].

Although Cu is an essential micronutrient for plant growth, it can be more toxic than nonessential Pb to biota when extraneous Cu is present in soil environments. Plant contents for Cu above 25 mg kg^−1^ are considered toxic to plants [25]. According to this criterion, investigated regions are not highly polluted by copper since its content in all plant species did not exceed the upper limit.

Nickel as a heavy metal belongs to a group of essential microelements to plants, animals, and humans, and its amounts exceeding optimum values show a toxic effect. Ni contents in plants range from 0.5 to 5 mg kg^−1^ dry weight and the values exceeding these limits are reported as toxic [25]; so in respect of the fact that Ni-uptake relies upon plant species and that some of plants show hyperaccumulation effects, investigated plant do not belongs in this group.

The results above indicated that the investigated plant contains large amounts of essential metals, and again plants sampled from Veles area are rich in this metal compared with control samples. The abundance of Mg, Ca, and Na in the result of this analysis, was in agreement with previous findings that these three elements represent the most abundant metal constituents in plants [26, 27].

The ability of plants to increase antioxidative protection to combat negative consequences of heavy metal stress appears to be limited since many studies showed that exposure to elevated concentrations of redox reactive metals resulted in decreased and not in increased activities of antioxidative enzymes. This fact is also valid for *U. dioica*, as shown in studies of many authors [13, 28, 29]. As FRAP assay measures only nonenzymatic (reductants) antioxidants in the sample, there is an interesting relationship among metal content and obtained FRAP value, valid for all investigated metals which are redox metals. Results obtained from FRAP assay in this study show that heavy metals induces oxidative stress in experimental model system which is evident from antioxidant levels (in *μ*mol FeSO_4_ L^−1^), as in all cases levels for total antioxidant activity in samples exposed to metals are lower than total antioxidant level in control sample. Antioxidant systems and their significance for the acclimation of plants to air pollution and climatic stresses have been reviewed frequently with emphasis on the responses of leaves [30–32]. Exposure to heavy metals also provoked responses of antioxidative systems, but the direction of response is dependent on the plant species, tissue analyzed, the metal used for treatment, and also intensity of the metal stress [2, 33]. However, some common reaction patterns can be found, for example, decreasing activities of antioxidative enzymes after metal exposition. In most cases, exposure to heavy metals, Cd and some others as Cu, Ni, and Zn, initially resulted in a severe depletion of glutathione (GSH), which is only one example. This is a common response to Cd caused by an increased consumption of GSH for phytochelatin production and their role in sequestering heavy metals which is a mechanism that contributes to the protection from metal toxicity in different plants and in some fungi as well [34]. In average, the samples exposed to metals in our study show for 61.9% lower antioxidant activity from the control sample (leaves) and 33.5% lower antioxidants level for the stems (Table 2). Evident are higher values for total antioxidants in plant leaves, which is in accordance with previously published results [35] where leaves from the plants are noted as plant organs richest with the antioxidants that prevent DNA damages induced by heavy metal stress.

RAPD technique, as PCR-based technique, has been successfully used to detect DNA damage and mutations in plants induced by various types of toxic chemicals [8, 9, 36]. Each fragment in RAPD is derived from a region of the genome that contains two short segments in inverted orientation on opposite strands that are complementary to the primer and sufficiently close together for the amplification process [37]. Polymorphism observed in RAPD profiles included disappearance and/or appearance of bands in comparison with control samples that were evaluated (Figures 2 and 3).

The RAPD profiles obtained exhibited bands between 1250 and 5000 bp in length. In a total of 14 bands scored, 13 were polymorphic (92.86%); we scored total 1 band with primer 1; 1 with primer 2; 3 with primer 3; 2 with primer 4; 3 with primer 5; 1 with primer 6; 2 with primer 7; by means of statistics there, is an average of 1.86 bands per primer (Table 3). Amplification with primer 1 and primer 6, yielded only one fragment with each of the primers, and we find this primer sequence not suitable for fingerprinting Urtica's genome. 

The samples exposed to metals yielded a large number of new fragments (total 12) compared with only one disappeared fragment in the obtained RAPD profile. New RAPD amplification products may be related to mutations (new annealing events), large deletions (bringing to pre-existing annealing site closer), and/or homologous recombination (two sequences that match the sequences of primer) [7, 38]. The high number of new appeared bands that was observed in samples exposed to metals suggests that long-term exposition to metals in high doses probably cause mutations on genomic level in *U. dioica* plants. These unique bands clearly differentiated the samples exposed to heavy metal stress and would act as a marker for assessment of environmental exposition on metals.

Accordingly to the results, it may be noted that the nutrient imbalance leads to DNA damages, mutations on genomic level in case of *U. dioica,* and also effects plant antioxidative defense system which contributed to the toxic effects in plants exposed to the long-term high metal concentrations. 

## 4. Conclusions

Heavy metal stress can decrease total antioxidants level and induce DNA damage in *U. dioica*. The changes occurring in plants RAPD profiles following exposition to heavy metals can be successfully used as a sensitive tool for detecting metal-induced DNA damage and showed potential as a reliable assay for genotoxicity. The obtained results in this study suggested that the mineral nutrient imbalance, DNA damages, and decreased antioxidants levels were involved in the metal toxicity in *U. dioica* which may help to understand the mechanisms of metals genotoxicity in plants.

## Figures and Tables

**Table 1:** Elemental analysis of *U. dioica* sampled from two different areas (in mg kg^−1^ dry mass).

Plant organ investigated	Metals								
	Ca	Cd	Cu	Mg	Mn	Na	Ni	Pb	Zn
	Location (unpolluted area) Plačkovica Mountain								

*U. dioica* leaves Plačkovica	23281 ± 4	<LD*	6.73 ± 0.06	4295 ± 1.3	29.66 ± 0.08	52.33 ± 0.3	<LD	3.73 ± 0.1	14.24 ± 0.1
*U. dioica* leaves Veles	37725 ± 5	7.34 ± 0.04	11.3 ± 0.03	6412.4 ± 2.5	74.71 ± 0.14	138.1 ± 2.7	2.89 ± 0.04	102.2 ± 0.4	465.3 ± 0.6

	Location (polluted area) Veles								

*U. dioica* stems Plačkovica	9245 ± 5	<LD*	7.16 ± 0.06	3366 ± 1.4	15.16 ± 0.08	46.43 ± 0.5	<LD	<LD	22.27 ± 0.3
*U. dioica* stems Veles	17816 ± 16	6.67 ± 0.03	8.25 ± 0.03	5036 ± 1.2	24.63 ± 0.05	86.95 ± 0.8	<LD	24.79 ± 0.2	229.5 ± 1.3

*LD is limit of detection (0.01 mg kg^−1^).

**Table 2:** Total antioxidants level in *U. dioica* sampled from two different areas obtained with FRAP assay (in *μ*mol FeSO_4_ L^−1^).

Plant organ investigated	FRAP values
	Location (unpolluted area) Plačkovica Mountain

*U. dioica* leaves Plačkovica	4845.5 ± 7.3
*U. dioica* leaves Veles	1849.6 ± 2.5

	Location (polluted area) Veles

*U. dioica* stems Plačkovica	961 ± 1.9
*U. dioica* stems Veles	640 ± 1.1

**Table 3:** Changes in the RAPD profiles (the number of bands and molecular sizes—bp) related to metals exposition compared to control; “+” appearance of DNA bands and/or “−” disappearance of DNA bands for all primers in the *U. dioica* plants.

Plant	Primers						
	Primer 1	Primer 2	Primer 3	Primer 4	Primer 5	Primer 6	Primer 7
*U. dioica*, Plačkovica	0	2500	0	0	0	0	0
*U. dioica*, Veles	(+) 3000	(+) 0	(+) 2500; 2000; 1250	(+) 5000; 2500	(+) 5000; 4000; 3000	(+) 2000	(+) 3000; 2500
	(−) 0	(−) 2500	(−) 0	(−) 0	(−) 0	(−) 0	(−) 0
